# Whole body-MRI identifies widespread, low intensity inflammation in peripheral joints, and axial involvement in a third of patients with early, treatment-naïve, active PsA: data from the GOLMePsA clinical trial

**DOI:** 10.1093/rheumatology/keag308

**Published:** 2026-06-17

**Authors:** Gabriele De Marco, Walter P Maksymowych, Mikkel Østergaard, Iris Eshed, Ai Lyn Tan, Philip S Helliwell, Dennis McGonagle, Elizabeth M A Hensor, Helena Marzo-Ortega

**Affiliations:** NIHR Leeds Biomedical Research Centre, The Leeds Teaching Hospitals NHS Trust, Leeds, UK; Leeds Institute of Rheumatic and Musculoskeletal Medicine, University of Leeds, Leeds, UK; Department of Medicine, University of Alberta and CARE Arthritis, Edmonton, Alberta, Canada; Copenhagen Center for Arthritis Research, Center for Rheumatology and Spine Diseases, Rigshospitalet, Glostrup, and Department of Clinical Medicine, University of Copenhagen, Copenhagen, Denmark; Department of Diagnostic Imaging, Sheba Medical Center, affiliated to the Gray Faculty of Medicine and Health Sciences, Tel-Aviv University, Tel Aviv, Israel; NIHR Leeds Biomedical Research Centre, The Leeds Teaching Hospitals NHS Trust, Leeds, UK; Leeds Institute of Rheumatic and Musculoskeletal Medicine, University of Leeds, Leeds, UK; NIHR Leeds Biomedical Research Centre, The Leeds Teaching Hospitals NHS Trust, Leeds, UK; Leeds Institute of Rheumatic and Musculoskeletal Medicine, University of Leeds, Leeds, UK; NIHR Leeds Biomedical Research Centre, The Leeds Teaching Hospitals NHS Trust, Leeds, UK; Leeds Institute of Rheumatic and Musculoskeletal Medicine, University of Leeds, Leeds, UK; NIHR Leeds Biomedical Research Centre, The Leeds Teaching Hospitals NHS Trust, Leeds, UK; Leeds Institute of Rheumatic and Musculoskeletal Medicine, University of Leeds, Leeds, UK; NIHR Leeds Biomedical Research Centre, The Leeds Teaching Hospitals NHS Trust, Leeds, UK; Leeds Institute of Rheumatic and Musculoskeletal Medicine, University of Leeds, Leeds, UK

**Keywords:** whole body MRI, early PsA, treatment naïve, clinical trial, axial involvement

## Abstract

**Objectives:**

To describe the prevalence and extent of whole body (WB)-MRI detected joint disease features and their response to therapy in patients with early, active PsA.

**Methods:**

Newly diagnosed PsA patients (treatment-naïve), recruited in the GOLMePsA randomized trial (MTX plus golimumab and steroids, GOLMTX vs MTX plus steroids, PBOMTX), underwent a multi-joint MRI protocol (baseline; week 24 [primary outcome] and week 36). The validated WIPE (peripheral joints and entheses); HIMRISS, KIMRISS (hip and knee); HEMRIS (heel) and CANDEN and SPARCC (sacroiliac joints (SIJs), spine) scores were recorded. Exploratory estimates of difference or ratios (with CIs; 75–95%) between groups at weeks 24 and 36 were obtained using multiple binary logistic or quantile (median) regression.

**Results:**

Overall, 93 paired scans from 31 participants (median symptom duration 10.5 months; IQR 4.2–18.3; absolute range 1.8–197.7; 68% polyarticular) were included. Baseline scores showed widespread low intensity inflammation in peripheral tissues (median MRI-WIPE score: 37—CI 19.0–55.0). SIJs and spine inflammation (SPARCC score ≥2) was seen in 25.8% (10/31) and 32.3% (10/31), respectively. Participants achieved median MRI-WIPE delta (the difference between time points) of −10 at week 24 and −7 at week 36 in the GOLMTX and −6 and −9 in PBOMTX arms, respectively. No differences between the treatment groups were observed.

**Conclusions:**

In this exploratory analysis, WB-MRI identified widespread although low intensity inflammation in peripheral joints and enthesis with improvements seen at 24 and 36 weeks and no meaningful differences between treatment arms. Axial abnormalities were seen in one third of patients at baseline.

**Clinical trial registration:**

Clinicaltrials.gov; NCT04108468

Rheumatology key messages

- One third of this early PsA cohort had axial involvement on baseline WB-MRI, mostly asymptomatic.
- WB-MRI inflammatory scores improved with no meaningful differences between treatment arms.
- Larger, controlled studies are needed to fully assess the validity of WB-MRI in PsA.

## Introduction

PsA is a progressive, chronic inflammatory disease characterized by substantial heterogeneity in clinical presentation and course. Symptoms caused by inflammation of the entheseal [1], synovial, osseous and extra-capsular tissues at peripheral and spinal joints may present in isolation or in combination or in sequence. Notably, clinical symptoms at the time of disease onset are often subtle and non-specific, contributing to significant diagnostic delay when compared with RA [2]. Previous studies have highlighted the role of imaging, in particular US, in identifying subclinical inflammatory burden in newly diagnosed PsA [3], which helps to map out extent and severity of disease. Recent reports utilizing US and conventional MRI have shown a low association of joint tenderness in the hand with imaging signs of inflammation in PsA patients, particularly in those with high levels of self-reported pain [4]. Other MRI studies showed only partial resolution of bone marrow oedema (BMO) in peripheral joints following therapy [5]. However, the more intriguing data related to imaging are those indicating that PsA-related pathology can occur at musculoskeletal (MSK) locations not affected by clinically-detected symptoms, with evidence suggesting that advanced imaging can reveal the presence of inflammation in MSK tissues that goes undetected upon clinical evaluation [3], even in cases deemed to be in clinical remission [6].

Whole body MRI (WB-MRI) has been shown as a feasible imaging method in both clinical and research settings. In PsA, it provides comprehensive evaluation of peripheral and axial joints, entheses, extra-capsular tissues and bone in one sitting [7, 8], complementing clinical examination by identifying most of the pathological spectrum of disease including subclinical inflammation. Further, WB-MRI has been shown to feasibly assess treatment response in PsA [9] and peripheral SpA [10]. However, data are limited on the prevalence and range of lesions identified in peripheral and axial MSK locations by WB-MRI in treatment naïve PsA of short disease duration.

The optimal treatment strategy in newly diagnosed, early PsA remains elusive. Emerging data suggest the enhanced value of prompt, intensive intervention at the time of diagnosis in early PsA when compared with standard of care. The GOLMePsA clinical trial assessed whether the combination of MTX, golimumab (GOL) plus corticosteroids was superior to MTX plus corticosteroids in reducing disease activity in early, untreated PsA [11] showing no superiority of one treatment arm to the other with comparable improvements observed by Psoriatic ArthritiS Disease Activity Score (PASDAS) [12] across both the treatment groups at weeks 24 and 52 [13]. Given that both clinically inaccessible and asymptomatic imaging pathology is common in PsA, and the ability of WB-MRI to provide wide imaging coverage of MSK joint structures; we hypothesized that WB-MRI would show a greater degree of subclinical inflammation across peripheral and axial joints in early PsA reflected in a higher level of change in imaging scores than clinical scores after treatment.

In this report, we aimed to add to the body of evidence, describing the prevalence and extent of disease features (inflammatory and structural) detected by WB-MRI across peripheral and axial joints, extra-capsular tissues and entheses—as well as their response to treatment—in a subset of patients with newly diagnosed, treatment naïve PsA recruited to the GOLMePsA clinical trial [13].

## Methods

### Summary of study design, patients and interventions

The GOLMePsA trial protocol, procedures and results have been reported [11, 13]. Briefly, GOLMePsA was a double-blind, randomized, placebo-controlled, parallel group, single centre, 52-week clinical trial which recruited adults with newly diagnosed, treatment-naïve, active PsA. Inclusion criteria included ≤24 months disease duration and presence of active disease by either ≥3 swollen and tender joints or two swollen and tender joints plus entheseal tenderness at the calcaneum. At trial baseline (week 0), participants received methylprednisolone (120 mg intramuscular, single dose), commenced weekly MTX (increased to 25 mg within 28 days—forced titration) and were block randomized (1:1) to arm 1 (combination of GOL and MTX; GOLMTX) or arm 2 (placebo [PBO] and MTX; PBOMTX)—stratified by oligoarticular/polyarticular involvement. By week 24 (the GOL/PBO interruption point), additional methylprednisolone was permitted just once (totalling up to 240 mg exposure). The primary end point (week 24) was the difference from baseline in mean PASDAS score [11, 13]. Ethical approval was granted by the Health Research Authority Research Ethics Committee (National Research Ethics Service Committee East Midlands, Northampton; reference, 14/EM/0124). All patients provided informed written consent. EudraCT registration number 2013–004122-28.

### Whole body MRI

WB-MRI was acquired using a commercially available 3.0 Tesla Siemens Vida system (Magnetom Vida, Siemens Healthcare, Erlangen, Germany) scanner. Scans were performed at three time points as per protocol [11]: baseline (pre-treatment); week 24 (primary outcome) and week 36 (3 months post-trial intervention), within 10 days before or after the scheduled visit attendance. Participants with any contraindications to MRI remained eligible to the GOLMePsA trial. The members of the research team who conducted the trial related clinical assessments had no role in image acquisition or scoring, to avoid breaches of the blind trial design.

### WB-MRI protocol

A multi-joint MRI protocol was designed using T1-weighted fat saturated spin echo (SE) before and after IV Gadolinium contrast injection (Volume-Interpolated Breath-hold Examination [VIBE] DIXON) and fluid-sensitive sequences providing coverage of shoulders, spine (cervical, thoracic, lumbar), pelvis, wrists and hands, knees, ankles and feet. T2-weighted fat saturated and Short Tau Inversion Recovery (STIR) sequences were acquired (parameters specified in Supplementary Table S1). WB-MRI images were first assessed for incidental abnormalities by authorized MSK radiologists blinded to treatment allocation or study details. Findings unrelated to PsA and implicating safety concerns or conditions requiring clinical action (as per National Health Service [NHS] guidance) were reported to the research team.

### MRI scoring

WB-MR images were scored at the end of the trial by three expert readers (two rheumatologists and one radiologist) blinded to clinical characteristics, treatment allocation and date of scan utilizing the established CARE ARTHRITIS platform (https://www.carearthritis.com/) and validated scoring systems. Peripheral joints were scored utilizing the MRI-WB scoring system for inflammation (WIPE) which assesses synovitis, BMO and soft tissue inflammation in 83 joints and 33 entheses [14]; with each joint or enthesis scored from 0 to 3; Hip Inflammation MRI Scoring System (HIMRISS) [15], Knee Inflammation MRI Scoring System (KIMRISS) [16] for inflammatory lesions in hips and knees respectively and Heel Enthesitis MRI Score (HEMRIS) [17] for inflammation and damage at the heel entheses (Achilles’ tendon and plantar fascia insertions). Joints in the axial skeleton were scored utilizing the Spondyloarthritis Research Consortium of Canada (SPARCC) scoring system–SIJ [18] for the sacroiliac joints (SIJs) and SPARCC spine [19] and Canada–Denmark MRI scoring system (CANDEN) [20] for the spine.

Inter- and intra-reader reliability for the scorers has been previously reported [21]. Previous calibration has shown high reliability can be attained, with percentage of exact agreement ranging from 90–97% (WB-MRI peripheral joints, intra-reader), to 99–100% (WB-MRI axial joints, intra-reader), to 84–97% (WB-MRI peripheral joints, inter-reader), to 98–100% (WB-MRI axial joints, inter-reader). Both intra- and inter-reader data showed Cohen’s *κ* values indicative of moderate to good agreement [21]. Scoring was performed by individual readers according to anatomical areas with single scores utilized for analysis.

### Statistical analysis

The MRI data presented herein were defined *a priori* as exploratory endpoints of the GOLMePsA trial [13]. Exploratory estimates of differences or ratios between the groups at weeks 24 and 36, and a range of CIs (75%, 85%, 95%), adjusted for screening values of the outcome and poly/oligoarthritis status, were obtained according to data type and distribution. The analysis of continuous interval outcomes used multiple linear regression. Differences between the treatment group means are presented. Adjusted estimates of effect size were provided for binary endpoints using multiple binary logistic regression and odds ratios were calculated. Severely skewed outcomes for which an appropriate generalized linear model could not be identified were analysed using quantile (median) regression; differences between medians were presented.

Readers did not score specific images if quality deemed too low. Available case analysis is presented throughout, without imputation for missing data, as stipulated in the analysis plan; numbers included in each analysis are presented in Supplementary Material. All analyses were conducted in StataCorp 2023. Stata 18 Base Reference Manual. College Station, TX: Stata Press).

## Results

**Figure 1:**
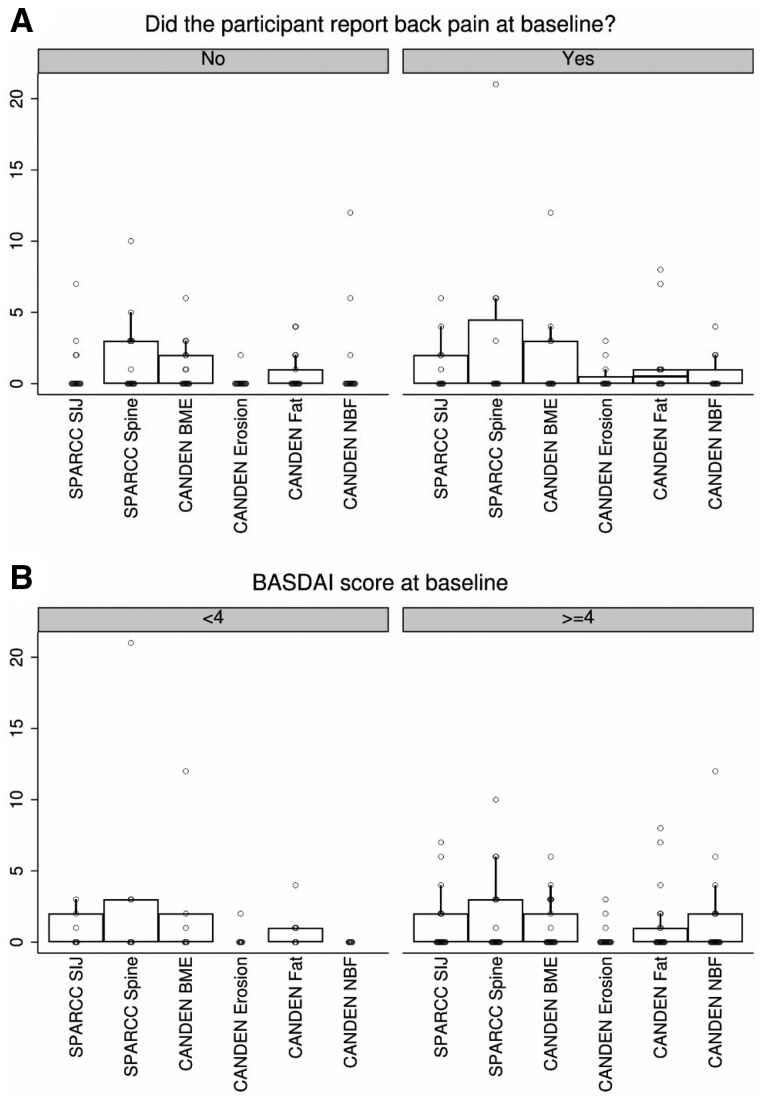
Axial MRI scores at baseline and patient-reported symptoms and outcomes. (**A**) Boxplot split by whether participant reported having back pain at baseline. (**B**) Boxplot split by whether participant scored <4/≥4 on BASDAI at baseline. Population: full analysis set (restricted to those with MRI data available). Graphical summary of descriptive data for axial scores. Observed data were plotted. BASDAI, Bath Ankylosing Spondylitis Disease Activity Index; BMO, bone marrow oedema; CANDEN, Canada–Denmark MRI scoring system; NBF, new bone formation; SPARCC, Spondyloarthritis Research Consortium of Canada

Between November 2015 and January 2018, 37 persons who consented to take part in GOLMePsA underwent the scheduled MRI scans that preceded exposure to trial interventions. Of these, two (2/37; 5.4%) failed the screening procedures and hence concluded study participation prior to exposure to trial interventions (Fig. 1). By 2018, and half-way through study recruitment (49/84), MRI acquisition was stopped due to the unexpected decommissioning of the existing MRI scanner [22] (Supplementary Fig. S1). An amendment to protocol (v7.0—13/03/2019) [13] documented this event and the need for WB-MRI outcomes to be moved from secondary to exploratory.

In total, 35 participants had baseline MRI scans, representing 41.7% (35/84) of the total study population [13]. Of these, 31 (88.6%) had follow-up scans at weeks 24 and 36. Baseline clinical characteristics are presented in Table 1. No selection bias was seen in this population, as shown by the descriptive comparison of baseline characteristics between participants with and without MRI data (Supplementary Table S2). In all, a total of 93 paired scans (baseline, week 24 and week 36) from 31 participants were scored and are presented herein.

**Table 1:** Baseline clinical characteristics of GOLMePsA participants who underwent MRI scanning.

Variable	Allocation		
	GOLMTX	PBOMTX	Total
	*N* = 17	*N* = 14	*N* = 31
Age, in years	40.3 (9.0); 28.0–58.0, *n* = 14	45.8 (13.3); 18.0–65.0, *n* = 17	43.3 (11.7); 18.0–65.0, *n* = 31
Sex			
Male	9 (64.3%)	11 (64.7%)	20 (64.5%)
Female	5 (35.7%)	6 (35.3%)	11 (35.5%)
Ethnicity group			
White	7 (50.0%)	16 (94.1%)	23 (74.2%)
Not stated	7 (50.0%)	1 (5.9%)	8 (25.8%)
Psoriasis symptom duration, months	169.6 (47.8, 197.2); 10.1–234.4, *n* = 13	221.7 (69.2, 378.3); 0.7–502.8, *n* = 17	174.8 (60.1, 252.1); 0.7–502.8, *n* = 30
Psoriasis duration, months	62.5 (2.5, 169.6); 0.3–234.4, *n* = 13	209.7 (60.5, 366.3); 0.2–499.6, *n* = 17	87.2 (7.1, 252.1); 0.2–499.6, *n* = 30
Joint symptom duration, months	7.1 (4.1, 11.4); 1.8–36.7, *n* = 14	13.1 (9.3, 23.3); 1.9–197.7, *n* = 17	10.5 (4.2, 18.3); 1.8–197.7, *n* = 31
PsA disease duration, months	0.7 (0.3, 1.6); 0.0–3.7, *n* = 14	0.6 (0.2, 1.1); 0.1–4.9, *n* = 17	0.6 (0.3, 1.5); 0.0–4.9, *n* = 31
Family history of Psoriasis			
No	10 (71.4%)	7 (41.2%)	17 (54.8%)
Yes	4 (28.6%)	10 (58.8%)	14 (45.2%)
Family history of PsA			
No	12 (85.7%)	15 (88.2%)	27 (87.1%)
Yes	2 (14.3%)	1 (5.9%)	3 (9.7%)
Not known	0 (0.0%)	1 (5.9%)	1 (3.2%)
Family history of axSpA			
No	14 (100.0%)	15 (88.2%)	29 (93.5%)
Yes	0 (0.0%)	1 (5.9%)	1 (3.2%)
Not known	0 (0.0%)	1 (5.9%)	1 (3.2%)
Family history of IBD			
No	13 (92.9%)	14 (82.4%)	27 (87.1%)
Yes	1 (7.1%)	2 (11.8%)	3 (9.7%)
Not known	0 (0.0%)	1 (5.9%)	1 (3.2%)
Smoker			
Current	3 (21.4%)	3 (17.6%)	6 (19.4%)
Previous	3 (21.4%)	5 (29.4%)	8 (25.8%)
Never	8 (57.1%)	9 (52.9%)	17 (54.8%)
Pack years smoking	0.0 (0.0, 3.8); 0.0–13.0, *n* = 14	0.0 (0.0, 3.8); 0.0–30.0, *n* = 17	0.0 (0.0, 3.8); 0.0–30.0, *n* = 31
Dactylitis			
Current	11 (78.6%)	9 (52.9%)	20 (64.5%)
None	2 (14.3%)	7 (41.2%)	9 (29.0%)
Previous history	1 (7.1%)	1 (5.9%)	2 (6.5%)
Entheseal tenderness			
Absent	4 (28.6%)	10 (58.8%)	14 (45.2%)
Present	10 (71.4%)	7 (41.2%)	17 (54.8%)
Peripheral joints classification			
Oligoarthritis	5 (35.7%)	5 (29.4%)	10 (32.3%)
Polyarthritis	9 (64.3%)	12 (70.6%)	21 (67.7%)
Back pain			
Absent	7 (50%)	12 (70.6%)	19 (61.3%)
Present	7 (50%)	5 (29.4%)	12 (28.7%)
Axial disease (clinical)			
No	13 (92.9%)	17 (100.0%)	30 (96.8%)
Yes	1 (7.1%)	0 (0.0%)	1 (3.2%)
Psoriasis BSA			
BSA affected by psoriasis (%)	1.0 (0.5, 3.0); 0.0–48.0, *n* = 14	1.0 (0.5, 2.0); 0.0–20.0, *n* = 17	1.0 (0.5, 3.0); 0.0–48.0, *n* = 31
Psoriatic nail dystrophy			
No	5 (35.7%)	4 (23.5%)	9 (29.0%)
Yes	9 (64.3%)	13 (76.5%)	22 (71.0%)
Elevated CRP (≥5 mg/l)	8 (67.1%)	9 (53.0%)	17 (54.8%)
HLA B27 status			
Negative	10 (71.4%)	10 (58.8%)	20 (64.5%)
Positive	3 (21.4%)	2 (11.8%)	5 (16.1%)
Missing	1 (7.2%)	5 (29.4%)	6 (19.4%)
Anti-CCP			
Negative	12 (85.7%)	15 (88.2%)	27 (87.1%)
Positive	1 (7.1%)	1 (5.9%)	2 (6.5%)
Missing	1 (7.1%)	1 (5.9%)	2 (6.5%)
RF (IgM)			
Negative	13 (92.9%)	15 (88.2%)	28 (90.3%)
Positive	1 (7.1%)	1 (5.9%)	2 (6.5%)
Missing	0 (0.0%)	1 (5.9%)	1 (3.2%)
PASDAS score	5.8 (1.7); 2.6–8.7, *n* = 14	5.5 (1.3); 3.3–7.7, *n* = 16	5.6 (1.5); 2.6–8.7, *n* = 30

Categorical variables presented as *n* (%); continuous variables presented as mean (S.D.) or median (first quartile to third quartile); range, *n*.

GOLMTX, intervention arm 1, that is, combination of golimumab and MTX; PBOMTX, intervention arm 2, that is, combination of placebo and MTX; axSpA, axial SpA: IBD, inflammatory bowel disease; BSA, body surface area; CCP, cyclic citrullinated peptides; IgM, immuniglobulin M; PASDAS, Psoriatic ArthritiS Disease Activity Score.

### Baseline MRI findings

Data from the 31 basal scores showed (Table 2) median MRI-WIPE score of 37 with observed values ranging between 5.0 and 116.0 in peripheral joints. Low level inflammation scores were reported at the hips (median HIMRISS BMO score 0.0—range 0.0–0.0; HIMRISS effusion median score 6.0—range 0.0–20.0) and knees (median KIMRISS BMO score 5.5—range 0.0–66.0). With regards to calcaneal entheses, the median basal HEMRIS inflammation score was 1.0 (range 0.0–11.0), while the median HEMRIS structural score was 0.0 (range 0.0–0.0). Although swollen joint count and WIPE synovitis score were weakly correlated at the patient level at baseline (both Spearman rho ∼0.3), this was not seen at the joint level where MRI was more often reported as positive suggesting subclinical inflammation (Supplementary Tables S3 and S4, and Supplementary Fig. S2). No differences were seen in MRI scores by polyarthritis/oligoarthritis status (Supplementary Fig. S3).

**Table 2:** Whole body MRI (exploratory outcomes) total scores at baseline

Variable	Allocation		
	Arm 1 (GOLMTX) *N* = 14	Arm 2 (PBOMTX) *N* = 17	Total *N* = 31
Peripheral			
MRI-WIPE (total inflammation)	29.0 (14.0–40.0); 12.0–84.0, *n* = 13	42.0 (27.0–63.0); 5.0–116.0, *n* = 16	37.0 (19.0–55.0); 5.0–116.0, *n* = 29
HIMRISS BML	0.0 (0.0–0.0); 0.0–0.0, *n* = 14	0.0 (0.0–0.0); 0.0–0.0, *n* = 17	0.0 (0.0–0.0); 0.0–0.0, *n* = 31
HIMRISS effusion	5.0 (2.0–7.0); 0.0–14.0, *n* = 14	7.0 (4.0–11.0); 0.0–20.0, *n* = 17	6.0 (3.0–10.0); 0.0–20.0, *n* = 31
KIMRISS BML	3.0 (0.0–18.0); 0.0–32.0, *n* = 13	7.0 (0.0–25.0); 0.0–66.0, *n* = 17	5.5 (0.0–20.0); 0.0–66.0, *n* = 30
HEMRIS inflammation	1.5 (1.0–5.0); 0.0–7.0, *n* = 14	1.0 (0.5–4.0); 0.0–11.0, *n* = 16	1.0 (1.0–5.0); 0.0–11.0, *n* = 30
HEMRIS structural	0.0 (0.0–0.0); 0.0–0.0, *n* = 14	0.0 (0.0–0.0); 0.0–0.0, *n* = 16	0.0 (0.0–0.0); 0.0–0.0, *n* = 30
Axial			
SPARCC sacro-iliac	0.0 (0.0–2.0); 0.0–6.0, *n* = 14	0.0 (0.0–1.5); 0.0–7.0, *n* = 16	0.0 (0.0–2.0); 0.0–7.0, *n* = 30
SPARCC spine	0.0 (0.0–3.0); 0.0–6.0, *n* = 14	0.0 (0.0–4.0); 0.0–21.0, *n* = 16	0.0 (0.0–3.0); 0.0–21.0, *n* = 30
CANDEN BMO	0.0 (0.0–2.0); 0.0–3.0, *n* = 14	0.0 (0.0–3.0); 0.0–12.0, *n* = 16	0.0 (0.0–2.0); 0.0–12.0, *n* = 30
CANDEN erosion	0.0 (0.0–0.0); 0.0–3.0, *n* = 14	0.0 (0.0–0.0); 0.0–2.0, *n* = 16	0.0 (0.0–0.0); 0.0–3.0, *n* = 30
CANDEN fat	0.0 (0.0–2.0); 0.0–8.0, *n* = 14	0.0 (0.0–1.0); 0.0–4.0, *n* = 16	0.0 (0.0–1.0); 0.0–8.0, *n* = 30
CANDEN NBF	0.0 (0.0–0.0); 0.0–4.0, *n* = 14	0.0 (0.0–0.0); 0.0–12.0, *n* = 16	0.0 (0.0–0.0); 0.0–12.0, *n* = 30

Variables presented as median (first quartile to third quartile), range and number.

MRI-WIPE, MRI Whole-Body Score for Inflammation in Peripheral Joints and Entheses in Inflammatory Arthritis; HEMRIS, Heel Enthesitis MRI Scoring System; HIMRISS, Hip Inflammation MRI Scoring System; BML, bone marrow lesion; BMO, bone marrow oedema; KIMRISS, Knee Inflammation MRI Scoring System; SPARCC, Spondyloarthritis Research Consortium of Canada; CANDEN, Canada–Denmark MRI scoring system; NBF, new bone formation.

The median axial lesion scores (SPARCC SIJ; SPARCC spine; CANDEN BMO/erosions/fatty lesions/new bone formation) were 0.0 with the largest range (0.0–21.0) observed for SPARCC spine. Median scores in the spine and peripheral joints tended to be slightly higher in those with polyarthritis, whereas HIMRISS effusion was slightly higher in those with oligoarthritis (Supplementary Fig. S4).

All the variables recorded were skewed in distribution. Overall, higher median inflammatory values scored (MRI-WIPE, HIMRISS, KIMRISS and HEMRIS inflammation) were seen at peripheral joints, extra-synovial tissues and entheses; and lower at axial skeleton (Table 2).

When variables were dichotomized (Supplementary Table S5), the disproportion between peripheral and axial scores persisted, as inflammatory lesions at baseline were observed in 93.5% by MRI-WIPE, 87.1% by HIMRISS (effusion); 58.1% by KIMRISS BMO and 77.4% by HEMRIS (Supplementary Table S5). The dichotomized scores capturing the presence of inflammatory lesions in peripheral joints and enthesis also show that lesions, albeit usually at a low level, were present in virtually all participants who were scanned. The HEMRIS structural score detected no lesions in 96.8% of scanned participants (100% with scores available).

With regards to the spine and SIJs, inflammatory features were found at baseline in 35.5% (SPARCC spine and CANDEN BMO scores) and 29% (SPARCC SIJ score) of participants. Structural lesions in the spine such as fat lesions (CANDEN) were observed in 35.5% of participants, erosions (CANDEN score) in 12.9% and new bone formation (CANDEN) in 19.4% at baseline. Axial MRI inflammation scores tended to be slightly higher in participants reporting back pain at baseline (Fig. 1A), although the differences were modest and with small differences between participants scoring <4 vs ≥4 on the Bath Ankylosing Spondylitis Disease Activity Index (BASDAI) questionnaire [23] (Fig. 1B).

### WB-MRI assessment of response to trial interventions

**Figure 2:**
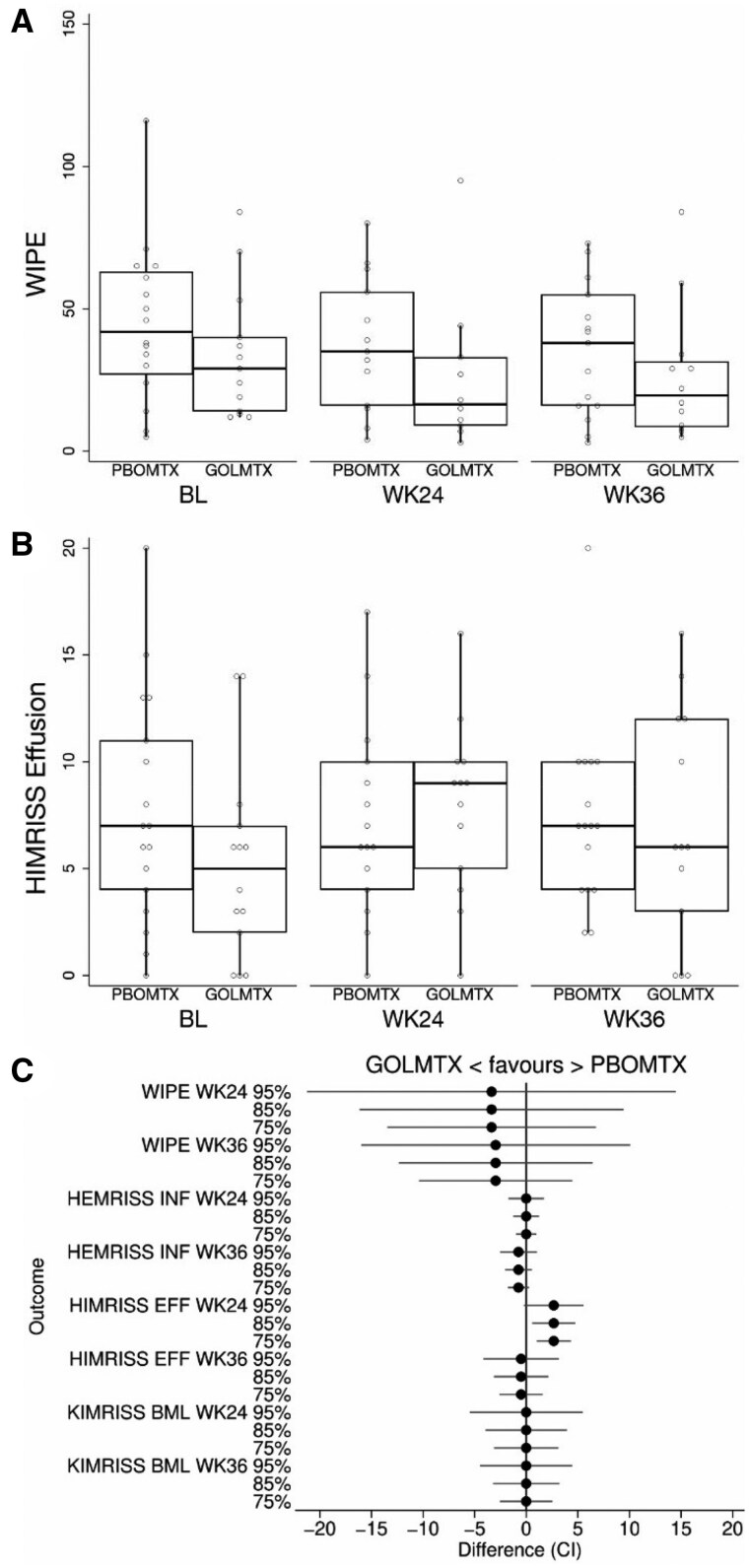
Differences between treatment arms over time. (**A**) Boxplot of MRI-WIPE, by treatment arm. (**B**) Boxplot of HIMRISS effusion, by treatment arm. (**C**) Forest plot of differences in exploratory MRI outcomes between treatment arms. Graphical summary of descriptive data for WIPE over time, by treatment arm. Observed data were plotted. Population: full analysis set (restricted to those with MRI data available). WIPE, MRI Whole-Body Score for Inflammation in Peripheral Joints and Entheses in Inflammatory Arthritis (MRI-WIPE); GOLMTX, combination of golimumab and MTX (arm 1); PBOMTX, combination of placebo and MTX (arm 2); BL, baseline; WK12, week 12; WK24, week 24; WK36, week 36; INF, inflammation; EFF, effusion; BML, bone marrow lesion

The analysis of follow-up MRI data showed improvements in the MRI-WIPE score at week 24 (Fig. 2A and C). Median delta (the difference between time points) of −10 at week 24 and −7 at week 36 and −6 (wk 24) and −9 (wk 36), were seen in the GOLMTX and PBOMTX arms respectively with no statistically significant differences between the two treatment arms, at any time point. CIs included 0 even at 75% confidence (week 24: −3.36; −13.53 to 6.81; week 36: −2.96; −9.94 to 4.02; see Supplementary Table S1). No participant achieved remission (total score = 0) by criterion of MRI-WIPE.

The HIMRISS effusion score apparently worsened in participants allocated to GOLMTX at week 24 (difference in medians 75% CI 1.02–4.32), only to revert to screening values at week 36 in the same group (75% CI −2.59 to 1.59) (Fig. 2B and C). The KIMRISS (BMO component) median delta at week 24 was 0.0 in both treatment arms. Median changes were −1 and 0 in the GOLMTX and PBOMTX groups, respectively, at week 36. There were no significant differences between treatment groups and all CIs included 0. The median HEMRIS inflammation score had little scope to improve as screening scores were low; median changes were both 0 at week 24 and −1 and 0 in the GOLMTX and PBOMTX groups, respectively, at week 36. There were no significant differences between the treatment groups and all CIs included 0. HEMRIS structural scores >0 were not present in either group at baseline.

Changes in CANDEN scores for the spine and SPARCC scores for spine and SIJs were sparse and required dichotomization. More participants had CANDEN BMO scores >0 in the PBOMTX arm (7/16) compared with GOLMTX arm (4/14) at baseline; the score improved in one GOLMTX participant at week 24, and two at week 36; with no improvements in PBOMTX participants. SPARCC MRI spine scores, which were balanced at baseline, improved in one participant in each arm at week 24 and in two GOLMTX participants at week 36, with no further improvements in PBOMTX participants. SPARCC MRI SIJs score improved in two GOLMTX participants and one PBOMTX participant at week 24, and three PBOMTX and one GOLMTX at week 36. For all dichotomized variables, the CIs around the odds ratios between the treatment groups were extremely wide and all included 1 at both time points (see Supplementary Table S2). Scores of CANDEN fat lesions and new bone formation did not show changes in either treatment group, at any time point.

## Discussion

**Figure 3:**
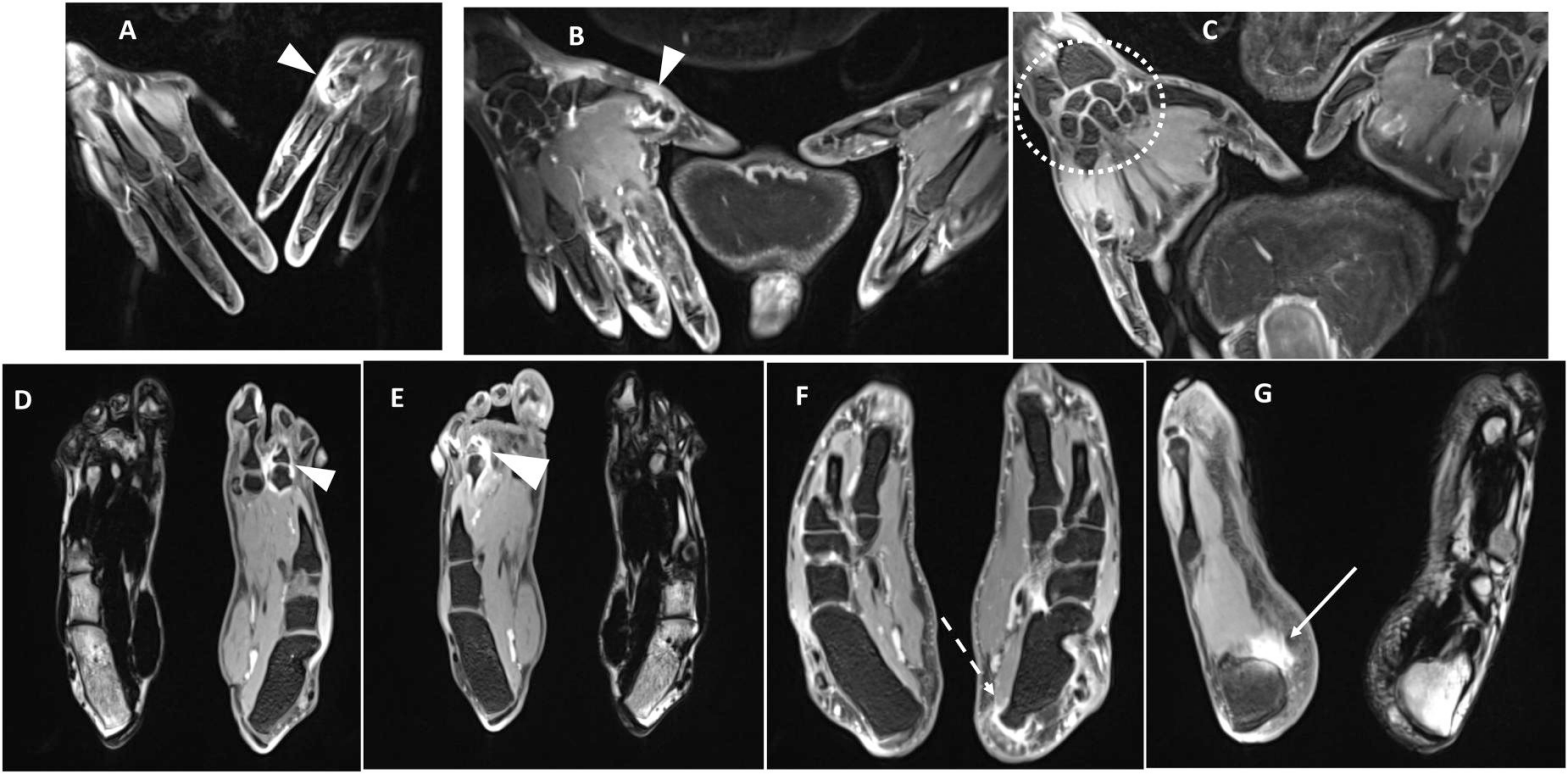
Examples of inflammatory findings in upper and lower limbs on WB-MRI. Figure shows examples of inflammatory findings on 3D VIBE Dixon post-Gadolinium images of the hands (coronal plane) and feet (axial plane) of different participants at the baseline study visit. Panels **A**, **B** and **C** illustrate findings of synovitis of metacarpo-phalangeal joints (Panels **A** and **B**, white arrowheads) and mild synovitis of the right wrist (Panel **C**, dashed white circle), with no findings in the left wrist. On images of the feet, synovitis of the metatarso-phalangeal joints (Panels **D** and **E**, white arrowheads), enthesitis at the distal insertion of the Achilles’ tendon onto the left calcaneum (Panel **F**, dashed white arrow) and enthesitis at the insertion of the plantar fascia onto the right calcaneum (Panel **G**, white arrow. Appearances on left calcaneum are due to artefact caused by failure of fat suppression) are seen

**Figure 4:**
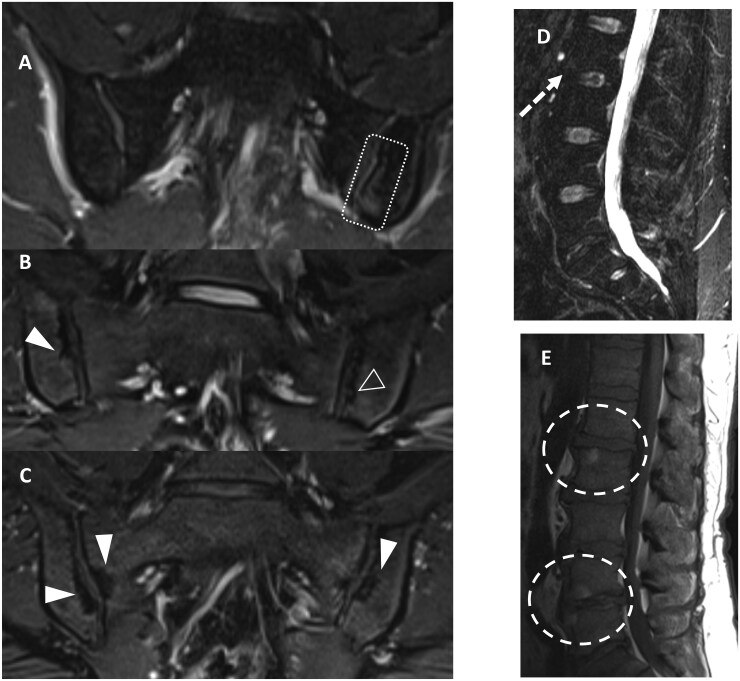
Examples of inflammatory and structural lesions in the sacroiliac joints and spine on WB-MRI. Figure illustrates examples of inflammatory and structural lesions in the sacroiliac joints and spine of different study participants at the baseline visit. Panels **A** and **B** are T2-weighted fat suppressed images acquired in the coronal oblique plane illustrating subchondral bone marrow oedema in the inferior aspect of the left sacroiliac joint (Panel **A**, dotted rectangle). Panel **B** illustrates subchondral sclerosis (white arrowhead) on the right and erosion in the left sacroiliac joint (white hollow arrowhead). Panel **C** is a T1-weighted image in the coronal oblique plane illustrating subchondral sclerosis (white arrowheads) in both sacroiliac joints. In Panel **D** mild bone marrow oedema (dotted arrow) is seen at the lower anterior corner of the L2 vertebra on the sagittal STIR sequence. Panel **E** is a T1-weighted sagittal image illustrating fat lesions in the upper anterior corners of L2 and L5 and the lower anterior corner of L4 (dotted circles)

This exploratory analysis aimed to describe the prevalence and extent of WB-MRI detected features of active and structural disease burden and their response to treatment in patients with early, treatment naïve PsA recruited to the GOLMePsA strategy clinical trial [13]. The results show widespread subclinical inflammation but with relatively modest scores in peripheral joints and peripheral entheses at baseline on WB-MRI, as well as a notable proportion of cases (about a third) with inflammatory lesions in the spine and/or SIJs, in this newly diagnosed PsA population (Figs 3 and 4). An improvement in WB-MRI lesions was seen at week 24, particularly in peripheral joints, independent of treatment allocation, mirroring clinical improvements in disease activity in both the treatment groups. Although the reported numbers are small, and a placebo treatment arm are lacking, these findings add to the understanding of inflammatory joint burden in newly diagnosed, untreated PsA of short symptom duration.

A strength of this *post hoc* analysis is the use of validated MRI scoring systems across both peripheral and axial domains in a well characterized early PsA cohort. However, there are several considerations when interpreting these data. First, it is important to highlight the eligibility criteria of the GOLMePsA trial [11] which emphasized the presence of active manifestations of PsA disease affecting primarily the peripheral joints and entheses. The median total MRI-WIPE score recorded was 37.0 (absolute range: 5.0–116.0) out of a possible 738 if all locations in the entire body were maximally inflamed. In absolute terms, this suggests that the overall ‘inflammatory burden’ detected by MRI in peripheral joints and entheses was widespread when compared with clinical examination, though in general it was of relatively modest intensity. However, the median MRI-WIPE score was slightly higher in this study compared with findings from the MOSAIC study [24] which reported an average MRI-WIPE score of 28.8 (S.D. 22.5) and comprised of a polyarthritis PsA population with prior exposure to up to two conventional synthetic DMARDs and an average disease duration of 1.9 years, i.e. longer than that of GOLMePsA recruits. Overall, the widespread inflammation detected on MRI in our study is in accordance with clinical findings, as >60% of GOLMePsA participants presented with polyarthritis and 65% had dactylitis, and consistent with previous observations published from the TICOPA study, also studying an early PsA population [25].

It is not possible, however, to comment on structural MRI lesions of peripheral joints/entheses in the dataset presented in this manuscript, since most scoring methods reported—i.e. MRI-WIPE, HIMRISS and KIMRISS do not capture structural lesions. By contrast, structural lesions (erosions, fat lesions and new bone formation) were detected by the axial evaluation score CANDEN (12.9%, 35.5% and 19.4%, respectively). Of note, however, was the absence of structural lesions in the heel entheses as detected by HEMRIS, which may be related to the short symptom duration of this study cohort.

One interesting finding is the prevalence of axial involvement reflected by one third of patients having MRI findings of active inflammation as shown by BMO lesions, slightly more frequent in the spine (35%) than in the SIJs (29%) in this newly diagnosed PsA population. Axial MRI scores were slightly higher in those reporting back pain and those with BASDAI >4. However, it is difficult to relate the patient-reported outcomes to the imaging findings since BASDAI is influenced by peripheral disease [26] and to investigate this properly, it would require a larger dataset and full adjustment for confounding of the relationship between MRI abnormalities and pain. These findings, nevertheless, are consistent with those reported in an earlier trial of patients with very early (<3 months symptom duration) peripheral SpA, of whom ∼40% were early PsA [27]. In this study, MRI sacroiliitis was detected in ∼30%, with just 10% reporting back pain although no spinal imaging was performed [27]. Similar prevalences of MRI inflammatory lesions in the spine and SIJs were reported in the AXIS study, with a PsA population of longer symptom duration (mean ± S.D. of 4.1 ± 2.9 years) [28] although slightly lower than that reported in a previous study of PsA with established disease [29]. Nevertheless, the prevalence of key spinal structural lesions, such as erosions and new bone formation, reported in the CANDEN score in our study (35% of participants) is of interest as it raises the question as to whether inflammatory involvement in the spine may precede peripheral joint symptoms. Nevertheless, no structural scores are available for the peripheral joints, the extra-capsular tissues, the entheses (with the notable exception of the calcaneus) and the SIJs in these data set, so these findings need external replication in larger cohorts. If confirmed, they would point to the need to consider the spine as a possible point of inflexion in some patients in the transition to PsA in the at-risk psoriasis population.

The lack of a consensus understanding on axial involvement in PsA is reflected by the wide prevalence estimates (12.5–78%) available in the current literature [30] that reflect the heterogeneity of definitions across different studies which varied from clinically based assessment to detection of axial skeleton pathology by imaging, including conventional radiography or CT or MRI. In the GOLMePsA trial, conventional radiography of pelvis and lumbar spine and HLA-B27 testing were not part of the scheduled procedures performed at screening/baseline. Further, clinical assessors were kept blinded to the results of the MRI scores until the time of statistical analysis of the data. The lack of access to any relevant imaging may have led to the under-representation of participants classified as ‘PsA with axial involvement’ by clinical means.

There are several limitations when interpreting the data from this exploratory analysis. Firstly, the acquisition protocol reflected practice at the time of trial inception, when more efficient sequences, i.e. gradient echo, were not routinely utilized. Secondly, when considering our description of lesion prevalence at baseline, it should be noted that this PsA population was primarily polyarticular with high the levels of disease activity at study entry. Therefore, it could be argued that the GOLMePsA cohort is not fully representative of an unselected early, untreated PsA population. Perhaps more relevant is the lack of a control population, needed to fully appreciate the significance of the low-grade inflammatory lesions seen, particularly in the axial skeleton. A recent imaging study in juvenile inflammatory arthritis (JIA) by Choida *et al.* reported inflammatory findings on WB-MRI in 15% of healthy controls uncovering subclinical inflammation in 40% of participants with JIA [31]. Of note, this study utilized post contrast coronal gradient echo Dixon images which may be more sensitive for the detection of inflammation particularly in peripheral and small joints than non-contrast MRI techniques, such as diffusion-weighted imaging or short-tau inversion recovery (STIR), more suited to assess BMO and used in our study [31]. As such, the data reported in the Choida *et al.* study [31] are highly relevant to understand the validity of WB-MRI in joint assessment in JIA with similar studies needed in PsA where data remain sparse [21].

Our results reflect the ability of WB-MRI to detect treatment response in PsA, as reflected in the WIPE scores. Indeed, when assessing the changes in WB-MRI scores on follow-up, a trend in decreased MRI-WIPE inflammatory lesions, regardless of treatment allocation was seen at 24 weeks, reflecting improvements seen in clinical response in both the groups. Notably, the primary outcome measure (PASDAS) [12] levels, which were >5 on average at baseline, improved in both treatment arms at week 24 [unadjusted mean (S.D.) GOLMTX: 2.70 (1.38); PBOMTX: 3.09 (1.32); baseline- and poly/oligoarthritis-adjusted difference (95% CI): −0.55 (−1.12 to 0.03); *P* = 0.064] [13]. Also, in both the treatment groups ∼55% achieved minimal disease activity at the different endpoints [13], although residual inflammatory lesions (BMO) were found on imaging at the end of the trial interventions (week 24) in some patients, irrespective of allocation or MDA status. The same, however, was not observed with SPARCC scores, that is, this axial score did not show detectable changes. The significance of the (minimal) residual inflammatory burden detectable on imaging in persons who achieved clinical improvement upon treatment is uncertain, and warrants consideration in larger, longitudinal data sets. Ideally, a third treatment arm with a placebo control group would be needed to fully discern the biological validity of WB-MRI lesions in our study. Other interventional studies have recently utilized WB-MRI in a PsA [24] and other peripheral SpA populations [10], showing the utility of different WB-MRI protocols in assessing treatment response with good sensitivity to change. Although like ours, none of these studies reported on a placebo-controlled imaging arm, earlier interventional studies in axial SpA showed good discrimination of a WB-MRI total inflammation index between the treatment and placebo groups [32, 33].

Considering these findings, our original hypothesis that WB-MRI would show a greater degree of subclinical inflammation across peripheral and axial joints in early PsA reflected in a higher level of change in imaging than clinical scores after treatment could not be unequivocally proven. For example, new MRI lesions were identified on follow-up scans, both in peripheral and axial joints which may or may not reflect biologic disease. In this light, further research is needed to fully elucidate the real value of WB-MRI protocols vs dedicated conventional MRI when assessing specific anatomical areas. Indeed, a recent review [34] reported some studies showing moderate to high correlation for BMO in SIJ and spine between a dedicated MRI scan and WB-MRI in SpA patients in one study [33] and another study showing similar results for the SIJ, but weaker or not significant correlations for spinal activity and damage [8]. Overall, a significant research gap remains in the absence of a reference standard of the full breath of inflammatory and structural lesions captured by WB-MRI in peripheral and axial joints in PsA in comparison with health, which is needed to fully evaluate the clinical relevance of our findings and to what extent these may reflect the presence or co-occurrence of non-inflammatory or mechanical pathology alongside real inflammatory burden.

In conclusion, in this small exploratory analysis, WB-MRI identified clinical and subclinical inflammation—albeit with relatively low scores in peripheral joints, enthesis and extra-capsular tissues at baseline—in a subset of participants in the GOLMePsA clinical trial. The change in lesions, with reading blinded to time point, over time regardless of treatment allocation mirrored clinical improvements. A third of patients in this newly diagnosed, treatment naïve, early PsA population had MRI abnormalities in the axial skeleton at baseline. These findings add to current knowledge, by contributing to the understanding of the full extent of disease in newly diagnosed, active PsA, currently underappreciated, and help direct research efforts into the earlier stages of disease.

## Supplementary Material

keag308_Supplementary_Data

## Data Availability

The sponsor will consider reasonable requests for clinical trial data from qualified researchers with an appropriate and clearly defined scientific objective, following the publication of the primary results of the trial. Data considered for sharing can include anonymized aggregate clinical trial data not covered by relevant privacy legislation, clinical study reports, statistical analysis plan, informed consent forms and protocol. Researchers will be required to sign a Data Use Agreement before receiving study documents.
