# Supplementary material for: Whole body-MRI identifies widespread, low intensity inflammation in peripheral joints, and axial involvement in a third of patients with early, treatment-naïve, active PsA: data from the GOLMePsA clinical trial
Source: Rheumatology (Oxford). 2026 Jun 17;65(7):keag308. doi: 10.1093/rheumatology/keag308 (PMC13332444; doi:10.1093/rheumatology/keag308)
Supplement: keag308_Supplementary_Data [file keag308_supplementary_data.docx]

# **De Marco G, et al. WB-MRI shows widespread inflammation in peripheral joints and one third axial involvement in early PsA: data from the GOLMePsA cl**i**nical trial**.

# **Supplementary Material**

## **Table S1. GOLMePsA Trial Magnetic Resonance Imaging Acquisition (MRI) Protocol**

| **Parts imaged** | **Coil** | **Sequence** | **FOV** | **Parameters (TR/TE/TI)** | **Resolution (mm)** | **Matrix** | **Slice gap** | **Time (mins)** | **Plane** |
| --- | --- | --- | --- | --- | --- | --- | --- | --- | --- |
| Shoulders | Large flex coil | STIR | 500 mm | 4500/101/220 | 1.7x1.3x4.0 | 288x384 voxels | 0.8 mm | 5:39 | Coronal |
| Spine (Cervical and thoracic) | Spine Coil | STIR  T1 | 450 mm | 4500/94/220  500/11 | 1.2x0.9x4.0  1.2x0.9x4.0 | 384x512 voxels | 0.4 mm | 3:02  3:01 | Sagittal  Sagittal |
| Spine (Thoracic and lumbar) | Spine Coil | STIR  T1 | 450 mm | 4500/94/220  500/11 | 1.2x0.9x4.0  1.2x0.9x4.0 | 384x512 voxels | 0.4 mm | 3:02  3:01 | Sagittal  Sagittal |
| Sacro-iliac joints | Spine coil | T2 FS  T1 | 240 mm | 4040/71  700/10 | 1.3x0.9x4.0  0.8x0.6x4.0 | 192x256 voxels  288x384 voxels | 0.4 mm | 3:44  2:17 | Coronal oblique  Coronal oblique |
| Hips | Body matrix | STIR | 450 mm | 4500/98/220 | 1.6x1.2x4.0 | 288x384 voxels | 0.8 mm | 5:39 | Coronal |
| Hands | Body matrix | 3D VIBE Dixon post Gadolinium | 430 mm | 6.36/2.45 & 3.7 | 0.8x0.8x0.8 (isotropic) | 512x512 voxels | N/A | 2:00 | Coronal |
| Knees | Body matrix | 3D VIBE Dixon post Gadolinium | 404 mm | 10/2.45 & 3.7 | 0.8x0.8x0.8 (isotropic) | 304x512 voxels | N/A | 3:28 | Transverse axis and Sagittal |
| Feet and ankles* | Head coil | 3D VIBE Dixon post Gadolinium | 420 mm | 10/2.45 & 3.7 | 0.8x0.8x0.8 (isotropic) | 384x512 voxels | N/A | 3:07 | Transverse axis and Sagittal |

The “parts imaged” column lists the selected anatomical regions, in order of acquisition.

Midfoot joints acquired: all tarsal joints; all tarsal-metatarsal joints. Hindfoot joints acquired: all talar-calcaneal joints. Ankle joints acquired: tibial-talar and fibular-talar joints.

FOV = Field Of View; TR = Time to Repetition; TE = Time to Echo; TI = Time to Inversion; STIR = Short Tau Inversion Recovery; FS = Fat Saturated; VIBE = Volumetric Interpolated Breath-hold Examination; N/A = Not Applicable.

## **Figure S1. Flow of GOLMePsA Participants Undergoing MRI Scanning**


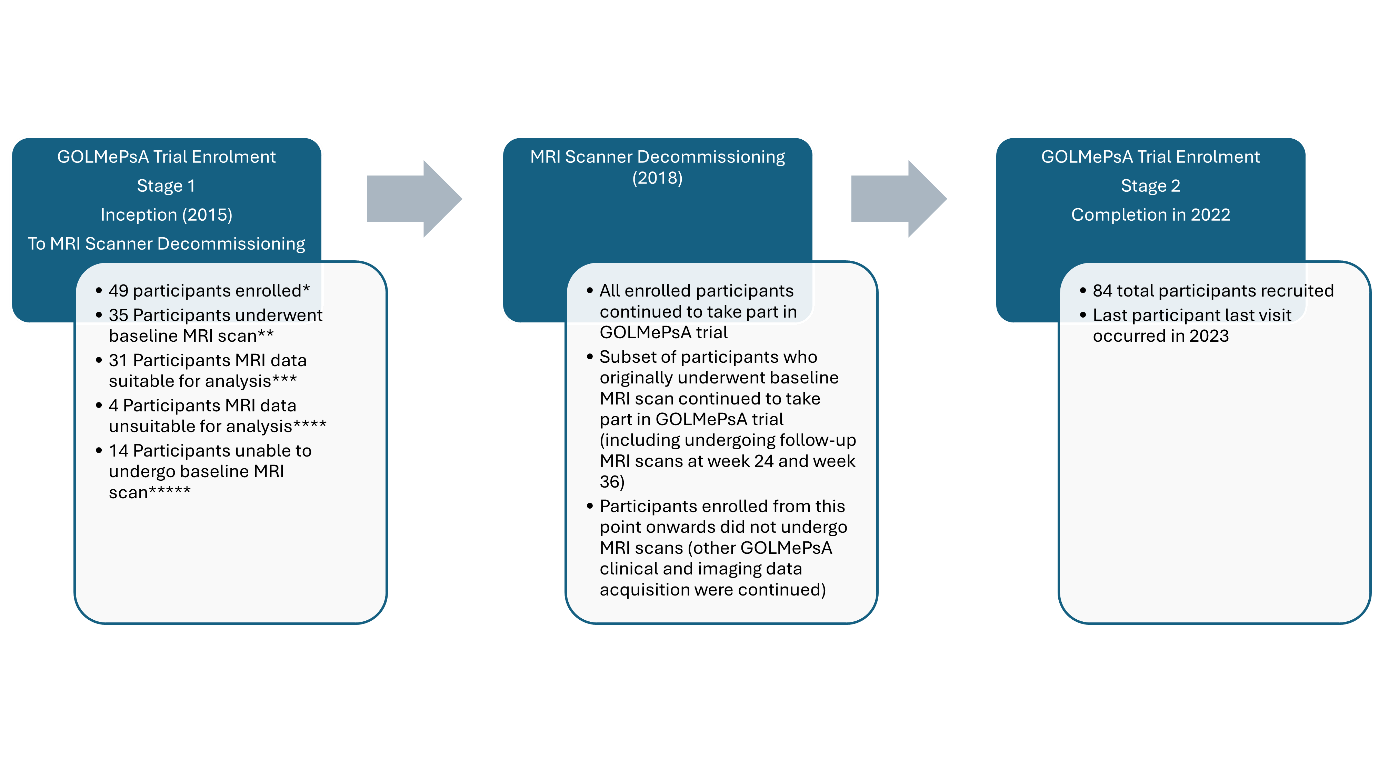


### **Table S2 - Baseline characteristics for GOLMePsA participants split by inclusion in MRI subset**

|  | **MRI subset** | |
| --- | --- | --- |
|  | **Not included** | **Included** |
|  | N=53 | N=31 |

| **Age (years)** | 42.0 (12.9); 23.0 to 73.0, n=53 | 43.3 (11.7); 18.0 to 65.0, n=31 |
| --- | --- | --- |
| **Sex** |  |  |
| Male | 26 (49%) | 20 (65%) |
| Female | 27 (51%) | 11 (35%) |
| **Ethnicity group** | |  |
| White | 38 (72%) | 23 (74%) |
| Not stated | 8 (15%) | 8 (26%) |
| **PsO symptom duration (mos)** | 124.4 (40.0, 287.9); 2.2 to 552.1, n=47 | 174.8 (60.1, 252.1); 0.7 to 502.8, n=30 |
| **PsO disease duration (mos)** | 80.8 (9.7, 229.9); 0.2 to 552.1, n=46 | 87.2 (7.1, 252.1); 0.2 to 499.6, n=30 |
| **Joint symptom duration (mos)** | 10.1 (5.3, 24.0); 1.7 to 61.5, n=53 | 10.5 (4.2, 18.3); 1.8 to 197.7, n=31 |
| **PsA disease duration (mos)** | 0.4 (0.2, 2.6); 0.1 to 7.7, n=53 | 0.6 (0.3, 1.5); 0.0 to 4.9, n=31 |
| **Family history of Pso** | |  |
| No | 26 (49%) | 17 (55%) |
| Yes | 26 (49%) | 14 (45%) |
| Not Known | 1 (2%) | 0 (0%) |
| **Family history of PsA** | |  |
| No | 44 (83%) | 27 (87%) |
| Yes | 8 (15%) | 3 (10%) |
| Not Known | 1 (2%) | 1 (3%) |
| **Family history of AS** | |  |
| No | 49 (92%) | 29 (94%) |
| Yes | 3 (6%) | 1 (3%) |
| Not Known | 1 (2%) | 1 (3%) |
| **Family history of IBD** | |  |
| No | 48 (91%) | 27 (87%) |
| Yes | 4 (8%) | 3 (10%) |
| Not Known | 1 (2%) | 1 (3%) |
| **Smoker** |  |  |
| Current | 10 (19%) | 6 (19%) |
| Previous | 17 (32%) | 8 (26%) |
| Never | 26 (49%) | 17 (55%) |
| **Pack years smoking** | 0.0 (0.0, 6.5); 0.0 to 41.2, n=51 | 0.0 (0.0, 3.8); 0.0 to 30.0, n=31 |
| **Dactylitis** |  |  |
| Current | 36 (68%) | 20 (65%) |
| None | 15 (28%) | 9 (29%) |
| Previous History | 2 (4%) | 2 (6%) |
| **Entheseal tenderness** | | |
| Absent | 18 (34%) | 14 (45%) |
| Present | 35 (66%) | 17 (55%) |
| **Oligo/polyarthritis (actual)** | | |
| Oligoarthritis | 13 (25%) | 10 (32%) |
| Polyarthritis | 40 (75%) | 21 (68%) |
| **Has back pain** | |  |
| No | 26 (49%) | 19 (61%) |
| Yes | 27 (51%) | 12 (39%) |
| **Axial disease** | |  |
| No | 51 (96%) | 30 (97%) |
| Yes | 2 (4%) | 1 (3%) |
| **BSA affected by Pso (%) BSL** | 0.9 (0.3, 2.8); 0.0 to 20.0, n=53 | 1.0 (0.5, 3.0); 0.0 to 48.0, n=31 |
| **PsO nail dystrophy** | |  |
| Not assessed | 2 (4%) | 0 (0%) |
| No | 23 (43%) | 9 (29%) |
| Yes | 28 (53%) | 22 (71%) |
| **HLA B27 status** |  |  |
| Positive | 13 (25%) | 5 (16%) |
| Negative | 36 (68%) | 21 (68%) |
| Not done | 4 (8%) | 5 (16%) |
| **C-reactive protein (mg/L)** | |  |
| <5 | 28 (53%) | 14 (45%) |
| 5-9.99 | 10 (19%) | 4 (13%) |
| >=10 | 15 (28%) | 13 (42%) |
| **Anti-CCP** | |  |
| Negative | 49 (92%) | 27 (87%) |
| Positive | 4 (8%) | 2 (6%) |
| Missing | 0 (0%) | 2 (6%) |
| **Rheumatoid factor** |  |  |
| Negative | 49 (92%) | 28 (90%) |
| Positive | 4 (8%) | 2 (6%) |
| Missing | 0 (0%) | 1 (3%) |
| **PASDAS** | 5.8 (1.1); 3.3 to 8.6, n=53 | 5.6 (1.5); 2.6 to 8.7, n=30 |

### **Comparison of baseline characteristics between MRI subset and remaining trial participants**

The 31 participants included in the MRI subset were recruited from the first 49 participants to be randomised to the GOLMePsA trial. Reasons for non-inclusion were: n=10 not eligible, n=4 unable to tolerate the scan, n=3 withdrawn from treatment, n=1 unable to attend. For the majority of the baseline characteristics those included in the MRI subset did not differ substantively from those excluded. A smaller proportion were female (35% vs 51%) and the duration of psoriasis symptoms was longer (median ~175 months vs ~124 months). However, psoriasis disease duration, and joint symptom/disease duration were similar between those included in or excluded from the MRI subset, as was the primary outcome PASDAS. Overall, there did not seem to be substantial differences that might suggest a possibility of bias in the results.

### **Table S3 - Agreement between MRI and clinical assessment**

This table presents sensitivity and specificity (with associated clustering-adjusted Wilson 95% confidence intervals) of clinical joint swelling, tenderness and swelling/tenderness (absent/present) for MRI BMO (bone marrow oedema) and MRI synovitis (score 0/>0) for peripheral joints at baseline, grouped by location.

|  | **MRI score** | |
| --- | --- | --- |
|  | **BME** | **Synovitis** |
| **Shoulders** |  |  |
| Swollen: Sensitivity | 0% [0/32] | 0% [0/32] |
| Swollen: Specificity | 100% [154/154] | 100% [154/154] |
| Tender: Sensitivity | 3.1 (0.5, 17.2) [1/32] | 12.5 (3.7, 34.5) [4/32] |
| Tender: Specificity | 85.1 (71.5, 92.8) [131/154] | 87.0 (73.0, 94.3) [134/154] |
| Swollen or tender: Sensitivity | 3.1 (0.5, 17.2) [1/32] | 12.5 (3.7, 34.5) [4/32] |
| Swollen or tender: Specificity | 85.1 (71.5, 92.8) [131/154] | 87.0 (73.0, 94.3) [134/154] |
| **Hands/wrists** |  |  |
| Swollen: Sensitivity | 54.3 (37.4, 70.2) [19/35] | 37.0 (28.3, 46.6) [54/146] |
| Swollen: Specificity | 91.4 (88.4, 93.7) [823/900] | 94.7 (92.1, 96.4) [747/789] |
| Tender: Sensitivity | 60.0 (45.2, 73.2) [21/35] | 45.2 (35.9, 54.9) [66/146] |
| Tender: Specificity | 82.6 (73.8, 88.8) [743/900] | 85.8 (76.1, 92.0) [677/789] |
| Swollen or tender: Sensitivity | 62.9 (50.0, 74.1) [22/35] | 47.3 (37.2, 57.5) [69/146] |
| Swollen or tender: Specificity | 81.7 (73.1, 88.0) [735/900] | 85.0 (75.5, 91.3) [671/789] |
| **Pelvis** |  |  |
| Swollen: Sensitivity | N/A (swelling not assessed) | N/A (swelling not assessed) |
| Swollen: Specificity | N/A (swelling not assessed) | N/A (swelling not assessed) |
| Tender: Sensitivity | 0% [0/2] | 0% [0/16] |
| Tender: Specificity | 100% [60/60] | 100% [46/46] |
| Swollen or tender: Sensitivity | N/A (swelling not assessed) | N/A (swelling not assessed) |
| Swollen or tender: Specificity | N/A (swelling not assessed) | N/A (swelling not assessed) |
| **Knees** |  |  |
| Swollen: Sensitivity | 28.6 (7.6, 65.9) [4/14] | 36.4 (14.0, 66.8) [8/22] |
| Swollen: Specificity | 77.8 (62.1, 88.2) [35/45] | 84.2 (69.4, 92.6) [32/38] |
| Tender: Sensitivity | 28.6 (8.3, 63.9) [4/14] | 40.9 (17.5, 69.3) [9/22] |
| Tender: Specificity | 62.2 (43.7, 77.8) [28/45] | 68.4 (47.5, 83.9) [26/38] |
| Swollen or tender: Sensitivity | 35.7 (12.2, 69.0) [5/14] | 45.5 (20.6, 72.8) [10/22] |
| Swollen or tender: Specificity | 55.6 (38.5, 71.4) [25/45] | 60.5 (41.4, 76.9) [23/38] |
| **Ankles/feet** |  |  |
| Swollen: Sensitivity | 22.6 (15.1, 32.3) [14/62] | 16.4 (10.4, 24.8) [32/195] |
| Swollen: Specificity | 93.1 (90.0, 95.3) [837/899] | 94.3 (91.3, 96.2) [723/767] |
| Tender: Sensitivity | 37.1 (25.2, 50.7) [23/62] | 30.3 (20.2, 42.6) [59/195] |
| Tender: Specificity | 82.8 (75.6, 88.1) [744/899] | 84.4 (76.6, 89.9) [647/767] |
| Swollen or tender: Sensitivity | 37.1 (25.2, 50.7) [23/62] | 30.3 (20.2, 42.6) [59/195] |
| Swollen or tender: Specificity | 81.5 (74.5, 87.0) [733/899] | 82.9 (75.3, 88.5) [636/767] |

MRI = Magnetic Resonance Imaging; BME = Bone Marrow Oedema; N/A = not assessed

### **Compatibility between WIPE and clinical scores:**

Any score >0 considered ‘present’ for comparison to clinical assessments. Where joints receiving just one assessment clinically had more than one score on WIPE, these were handled as follows:

- Upper sternoclavicular and lower sternoclavicular: maximum score taken for comparison to SCJ
- Intercarpal and carpometacarpal joints, distal radioulnar and radiocarpal: maximum score taken for comparison to wrist
- Posterior talocalcaneal and Talocrural: maximum score taken for comparison to ankle
- Talocalcaneonavicular and calcaneocuboid, and tarsal and tarsometatarsal: maximum score taken for comparison to tibio-tarsal

### **Table S4: Changes in MRI synovitis and clinical swelling at week 24**

|  |  |  | **Clinical swelling** | | | |
| --- | --- | --- | --- | --- | --- | --- |
|  | **Baseline** |  | **Absent** | | **Present** | |
|  |  | **Week 24** | **Absent** | **New** | **Resolved** | **Persistent** |
| **MRI** | **Absent** | **Absent** | 1,336 | 3 | 70 | 3 |
|  |  | **New** | 67 | 1 | 5 | 0 |
|  | **Present** | **Resolved** | 122 | 1 | 33 | 1 |
|  |  | **Persistent** | 150 | 1 | 39 | 8 |

Total of 1840 joints

- In joints which showed **neither clinical swelling nor MRI synovitis at baseline** (light blue quadrant), the majority (1336/1407) remained unaffected at week 24. However, 67 joints showed new MRI synovitis at week 24 in the absence of new clinical swelling (that is, developed subclinical synovitis at follow-up).
- In joints which were **clinically swollen at baseline in the absence of MRI synovitis** (n=78 -light orange quadrant), in the majority (n=70) the clinical swelling had resolved by week 24 and the MRI synovitis remained absent. In a minority, MRI synovitis had appeared even though the swelling had resolved (n=5), or the swelling remained in the absence of MRI synovitis (n=3).
- In joints that showed **MRI synovitis at baseline in the absence of clinical swelling** (subclinical synovitis at baseline – pink quadrant) 122/274 (45%) had resolved at week 24 without developing clinical swelling, while 150/274 (55%) persisted in the absence of swelling.
- In joints that showed **both clinical swelling and MRI synovitis at baseline** (grey quadrant), 8/81 (10%) persisted at week 24, whilst 39 (48%) showed subclinical synovitis, and 33 (41%) had entirely resolved.

These trends have been plotted in the Sankey plot (SM-Figure 2) available on the next page.

### **Figure S2 – Panel A (left) Sankey plot of trends in MRI changes; Panel B (right) correlation analysis SJC76 and WIPE at week 24**


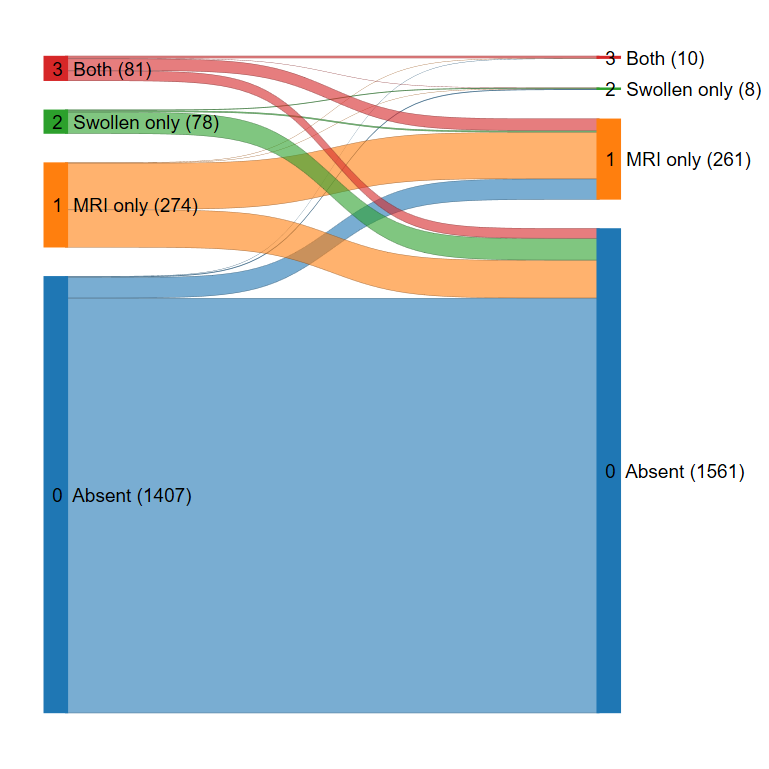

Panel A - Group 3 = Joints clinically swollen and with MRI synovitis. Group 2 = Joints clinically swollen, in absence of MRI synovitis. Group 1 = Joints not clinically swollen, but presence of MRI synovitis. Group 0 = Joints not clinically swollen and absence of MRI synovitis. SJC76 = swollen joint count. At the joint level this suggests that MRI synovitis is more responsive, appearing and disappearing first, before clinical swelling. However, at the participant level (panel B), although SJC76 and Whole-body MRI score for Inflammation in Peripheral joints and Entheses (WIPE) synovitis score (not analysed separately from BMO in the main MRI analysis, which only included total WIPE score) were weakly correlated at baseline (both Spearman rho~0.3), change in total WIPE synovitis score was not correlated with change in SJC76 between baseline at week 24 (Spearman rho=-0.06, n=23).

### **Figure S3 - MRI scores and poly/oligoarticular status**

Plot of Baseline MRI scores, by polyarthritis/oligoarthritis status. Median scores in the spine and peripheral joints tended to be slightly higher in those with polyarthritis, whereas HIMRISS effusion was slightly higher in those with oligoarthritis.

## **Table S5. Magnetic Resonance Imaging (Exploratory Outcomes), Dichotomized Scores at Baseline**

| **Variable** | **Allocation** | | |
| --- | --- | --- | --- |
|  | Arm 1 GOLMTX | Arm 2 PBOMTX | Total |
|  | N=14 | N=17 | N=31 |
| *Peripheral* |  |  |  |
| MRI-WIPE total inflammation score |  |  |  |
| >0 | 13 (92.9%) | 16 (94.1%) | 29 (93.5%) |
| 0 | 0 (0.0%) | 0 (0.0%) | 0 (0.0%) |
| Missing | 1 (7.1%) | 1 (5.9%) | 2 (6.5%) |
| HIMRISS BML |  |  |  |
| >0 | 0 (0.0%) | 0 (0.0%) | 0 (0.0%) |
| 0 | 14 (100.0%) | 17 (100.0%) | 31 (100.0%) |
| HIMRISS Effusion score |  |  |  |
| >0 | 11 (78.6%) | 16 (94.1%) | 27 (87.1%) |
| 0 | 3 (21.4%) | 1 (5.9%) | 4 (12.9%) |
| KIMRISS BML score |  |  |  |
| >0 | 7 (50.0%) | 11 (64.7%) | 18 (58.1%) |
| 0 | 6 (42.9%) | 6 (35.3%) | 12 (38.7%) |
| Missing | 1 (7.1%) | 0 (0.0%) | 1 (3.2%) |
| HEMRIS Inflammation score |  |  |  |
| >0 | 12 (85.7%) | 12 (70.6%) | 24 (77.4%) |
| 0 | 2 (14.3%) | 4 (23.5%) | 6 (19.4%) |
| Missing | 0 (0.0%) | 1 (5.9%) | 1 (3.2%) |
| HEMRIS Structural score |  |  |  |
| >0 | 0 (0.0%) | 0 (0.0%) | 0 (0.0%) |
| 0 | 14 (100.0%) | 16 (94.1%) | 30 (96.8%) |
| Missing | 0 (0.0%) | 1 (5.9%) | 1 (3.2%) |
|  |  |  |  |
| *Axial* |  |  |  |
| SPARCC Sacro-iliac joint inflammation score |  |  |  |
| >0 | 4 (28.6%) | 5 (29.4%) | 9 (29.0%) |
| 0 | 10 (71.4%) | 11 (64.7%) | 21 (67.7%) |
| Missing | 0 (0.0%) | 1 (5.9%) | 1 (3.2%) |
| SPARCC Spine inflammation score |  |  |  |
| >0 | 4 (28.6%) | 7 (41.2%) | 11 (35.5%) |
| 0 | 10 (71.4%) | 9 (52.9%) | 19 (61.3%) |
| Missing | 0 (0.0%) | 1 (5.9%) | 1 (3.2%) |
| CANDEN spine BMO score |  |  |  |
| >0 | 4 (28.6%) | 7 (41.2%) | 11 (35.5%) |
| 0 | 10 (71.4%) | 9 (52.9%) | 19 (61.3%) |
| Missing | 0 (0.0%) | 1 (5.9%) | 1 (3.2%) |
| CANDEN spine Erosion score |  |  |  |
| >0 | 2 (14.3%) | 2 (11.8%) | 4 (12.9%) |
| 0 | 12 (85.7%) | 14 (82.4%) | 26 (83.9%) |
| Missing | 0 (0.0%) | 1 (5.9%) | 1 (3.2%) |
| CANDEN spine Fat score |  |  |  |
| >0 | 6 (42.9%) | 5 (29.4%) | 11 (35.5%) |
| 0 | 8 (57.1%) | 11 (64.7%) | 19 (61.3%) |
| Missing | 0 (0.0%) | 1 (5.9%) | 1 (3.2%) |
| CANDEN spine NBF score |  |  |  |
| >0 | 3 (21.4%) | 3 (17.6%) | 6 (19.4%) |
| 0 | 11 (78.6%) | 13 (76.5%) | 24 (77.4%) |
| Missing | 0 (0.0%) | 1 (5.9%) | 1 (3.2%) |
| Categorical variables presented as n (%). MRI-WIPE = MRI Whole-Body Score for Inflammation in Peripheral Joints and Entheses in Inflammatory Arthritis; HEMRIS = Heel Enthesitis MRI Scoring; HIMRISS = Hip Inflammation MRI Scoring System; BML/BMO = Bone Marrow Lesion/Oedema; KIMRISS = Knee Inflammation MRI Scoring System; SPARCC = Spondyloarthritis Research Consortium of Canada; CANDEN = Canada-Denmark MRI Scoring System; NBF = New Bone Formation. | | | |

#### **Table S6: Sparse MRI outcomes; 95% CIs presented**

#### This table presents results for exploratory MRI outcomes which, due to sparsity in the data, needed to be coded as score improved (Y/N). This approach was unplanned. Descriptive data (n /N (%)) are presented for the observed values at baseline and the numbers and percentages of participants whose scores had improved at weeks 24 & 36. The odds ratio between the treatment arms has been calculated using Firth's penalised maximum likelihood logistic regression, due to data sparsity which caused the standard maximum likelihood binary logistic regression model to separate. Each estimate has been adjusted for baseline values of the outcome (without using binary coding) and the stratification variable poly/oligoarthritis status (using the participant's actual status). The total number of participants included in analysis at each visit, Ninc, is also presented. No analysis of CANDEN Fat, CANDEN SAS, HEMRIS structural or HIMRISS BML, at either 24 or 36 weeks, was attempted as there were no improvements in either treatment arm.

|  | PBOMTX | | | GOLMTX | | | OR | 95% | CI | t | p-value | Ninc |
| --- | --- | --- | --- | --- | --- | --- | --- | --- | --- | --- | --- | --- |
|  | n | N | % | n | N | % |  |  |  |  |  |  |
| CANDEN BMO binary BSL | 7 | /16 | (43.8) | 4 | /14 | (28.6) |  |  |  |  |  |  |
| CANDEN BMO WK24 Improved (Y/N) | 0 | /14 | (0.0) | 1 | /14 | (7.1) | 5.84 | (0.09, | 374.07) | 0.83 | 0.406 | 28 |
| CANDEN BMO WK36 Improved (Y/N) | 0 | /15 | (0.0) | 2 | /13 | (15.4) | 21.06 | (0.04, | 10881.51) | 0.96 | 0.339 | 28 |
| CANDEN Fat binary BSL | 5 | /16 | (31.2) | 6 | /14 | (42.9) |  |  |  |  |  |  |
| CANDEN Fat WK24 Improved (Y/N) | 0 | /14 | (0.0) | 0 | /14 | (0.0) |  |  |  |  |  |  |
| CANDEN Fat WK36 Improved (Y/N) | 0 | /15 | (0.0) | 0 | /13 | (0.0) |  |  |  |  |  |  |
| CANDEN Erosion binary BSL | 2 | /16 | (12.5) | 2 | /14 | (14.3) |  |  |  |  |  |  |
| CANDEN Erosion WK24 Improved (Y/N) | 1 | /14 | (7.1) | 1 | /14 | (7.1) | 0.80 | (0.06, | 10.38) | -0.17 | 0.864 | 28 |
| CANDEN Erosion WK36 Improved (Y/N) | 1 | /15 | (6.7) | 1 | /13 | (7.7) | 0.86 | (0.06, | 11.37) | -0.12 | 0.907 | 28 |
| CANDEN SAS binary BSL | 3 | /16 | (18.8) | 3 | /14 | (21.4) |  |  |  |  |  |  |
| CANDEN SAS WK24 Improved (Y/N) | 0 | /14 | (0.0) | 0 | /14 | (0.0) |  |  |  |  |  |  |
| CANDEN SAS WK36 Improved (Y/N) | 0 | /15 | (0.0) | 0 | /13 | (0.0) |  |  |  |  |  |  |
| HEMRIS Structural binary BSL | 0 | /16 | (0.0) | 0 | /14 | (0.0) |  |  |  |  |  |  |
| HEMRIS Structural WK24 Improved (Y/N) | 0 | /13 | (0.0) | 0 | /12 | (0.0) |  |  |  |  |  |  |
| HEMRIS Structural WK36 Improved (Y/N) | 0 | /15 | (0.0) | 0 | /12 | (0.0) |  |  |  |  |  |  |
| HIMRISS BML binary BSL | 0 | /17 | (0.0) | 0 | /14 | (0.0) |  |  |  |  |  |  |
| HIMRISS BML WK24 Improved (Y/N) | 0 | /15 | (0.0) | 0 | /13 | (0.0) |  |  |  |  |  |  |
| HIMRISS BML WK36 Improved (Y/N) | 0 | /16 | (0.0) | 0 | /13 | (0.0) |  |  |  |  |  |  |
| SPARCC SIJ binary BSL | 5 | /16 | (31.2) | 4 | /14 | (28.6) |  |  |  |  |  |  |
| SPARCC SIJ WK24 Improved (Y/N) | 1 | /14 | (7.1) | 2 | /13 | (15.4) | 2.18 | (0.21, | 22.69) | 0.65 | 0.515 | 27 |
| SPARCC SIJ WK36 Improved (Y/N) | 3 | /15 | (20.0) | 1 | /13 | (7.7) | 0.01 | (0.00, | 33.41) | -1.18 | 0.239 | 28 |
| SPARCC Spine binary BSL | 7 | /16 | (43.8) | 4 | /14 | (28.6) |  |  |  |  |  |  |
| SPARCC Spine WK24 Improved (Y/N) | 1 | /14 | (7.1) | 1 | /14 | (7.1) | 1.45 | (0.11, | 19.24) | 0.28 | 0.779 | 28 |
| SPARCC Spine WK36 Improved (Y/N) | 0 | /15 | (0.0) | 2 | /13 | (15.4) | 22.16 | (0.04, | 13189.03) | 0.95 | 0.342 | 28 |
